# Evaluation of the Percentage of Monocyte Subpopulations with TLR2 and TLR4 Expression About Selected Skin Functional Parameters in Patients with Acne Vulgaris—Cross-Sectional Study

**DOI:** 10.3390/jcm14186449

**Published:** 2025-09-12

**Authors:** Ewelina Firlej, Wioleta Grzegorzewska, Katarzyna Jastrzębska-Pawłowska, Mariola Janiszewska, Ilona Gąbka-Flis, Magdalena Makarska-Białokoz, Jacek Roliński, Joanna Bartosińska

**Affiliations:** 1 Department of Cosmetology and Aesthetic Medicine, Medical University of Lublin, 20-093 Lublin, Poland; ilona0002@gmail.com; 2 Department of Clinical Immunology, Medical University of Lublin, 20-093 Lublin, Poland; katarzyna.jastrzebska-pawlowska@umlub.edu.pl (K.J.-P.); jacek.rolinski@umlub.edu.pl (J.R.); 3 Department of Medical Informatics and Statistics with e-Health Lab, Medical University of Lublin, 20-059 Lublin, Poland; 4 Faculty of Health Sciences, Vincent Pol University in Lublin, 20-816 Lublin, Poland; mmakarska@wssp.edu.pl

**Keywords:** acne vugaris, skin, monocytes, TLR2, TLR4

## Abstract

**Background/Objectives:** Acne vulgaris can be non-inflammatory lesions, i.e., closed comedones, open comedones, inflammatory lesions, i.e., papules, pustules, cysts, and post-acne lesions. This study aimed to evaluate the expression of TLR2 and TLR4 receptors on classical, intermediate, and non-classical monocyte subpopulations in 38 women with acne vulgaris and to correlate the results with clinical features of the disease and selected skin parameters. **Methods:** The skin parameters were assessed: level of oiliness, hydration, pH, skin pigmentation (phototype, erythema) using a special diagnostic device (Scientific multi-probe system MPA 6, Courage + Khazaka) with simultaneous determination of monocyte subpopulations in peripheral blood expressing TLR2 and TLR4 using a CytoflexLX flow cytometer (Beckman Coulter). **Results:** In the study group, the percentage of non-classical monocytes expressing TLR2 was statistically significantly lower than the classical and intermediate monocytes expressing TLR2 (*p* < 0.001). However, the level of TLR2 receptor expression (MFI) was significantly higher on intermediate monocytes compared to the level of TLR2 expression on classical and non-classical monocytes. In the group of patients with post-acne lesions, a statistically significantly higher percentage of non-classical monocytes with TLR4 expression was observed compared to patients without post-acne lesions (*p* = 0.009). A statistically significant negative correlation was also observed between the percentage of intermediate and non-classical monocytes with TLR4 expression and the results of the mexameter measurements. Acne has a significant impact on the percentage of monocyte subpopulations expressing TLR2 and TLR4. A higher percentage of non-classical monocytes TLR4+ in the blood is associated with a higher incidence of post-acne lesions. **Conclusions:** The positive correlation between the degree of skin hydration and the level of TLR2 expression on classical monocytes suggests that these cells play an important role in skin homeostasis and defense against C. *acnes*. Proper acne care is not only important for aesthetic aspects, but may also have a positive impact on immunological phenomena.

## 1. Introduction

Acne vulgaris is an inflammatory disease involving the hair and sebaceous units. It is characterised by excessive sebum secretion, keratinisation of the hair follicle outlet and the presence of the Gram-positive anaerobic bacterium *Cutibacterium acnes* (formerly *Propionibacterium acnes*), and inflammation, which is the fourth process affecting the development of acne lesions [1,2,3,4]. Acne can be non-inflammatory lesions, i.e., closed comedones, open comedones, inflammatory lesions, i.e., papules, pustules, cysts, and post-acne lesions (erythema, hyperpigmentation, scarring) [5,6]. Increased sebum secretion, decreased hydration and a change in skin color are also characteristic features of acne-prone skin. The abnormalities described are diagnosed organoleptically both in clinical practice and in the cosmetologist’s office [7,8]. It is important to diagnose and start treating as soon as possible [9,10]. Treatment modalities include local and systemic therapy depending on the severity of the acne [11,12]. Therapy should be aimed at controlling inflammation and appropriately regulating the skin’s immune response [13,14]. Due to the recurrent nature of the disease, long-term supportive care is important [15]. In clinical practice, acne is one of the most common dermatological conditions. Its pathogenesis still requires further research and answers to many questions, which will undoubtedly contribute to improving the diagnosis and treatment of this disorder [11,16]. The aetiopathogenesis of the disease is multifactorial. Among the key mechanisms involved are changes in the fatty acid composition of the sebum [16]. Modifications of the composition of sebum may contribute to the occurrence of inflammation and initiate immunological mechanisms, leading to the occurrence and severity of acne lesions [17,18].

The overproduction of sebum in the sebaceous follicle promotes the proliferation of the bacterium *C. acnes* species and increases inflammation involving immune cells [19,20], including subpopulations of helper T lymphocytes [21,22,23], neutrophils [24,25], and monocytes/macrophages [26,27]. Monocytes are divided into subpopulations: CD14++ CD16- classical monocytes that migrate into tissues differentiate into M1 macrophages, and CD14+CD16+ monocytes differentiate into M2 macrophages [27]. CD16+ monocytes have been subdivided into an intermediate CD14++CD16+ phenotype and a non-classical CD14+/lowCD16++ phenotype [19]. Toll-like receptors (TLRs) are a family of eleven proteins that play a key role in the development of the innate immune response. Constituting a large subset of pattern recognition receptors (PRRs), they are responsible for recognising pathogen-specific structures known as pathogen-associated molecular patterns (PAMPs) or damage-associated molecular patterns (DAMPs) [28]. One of the main functions of TLRs is to activate mechanisms of antimicrobial activity and production of pro-inflammatory cytokines, including TNF, IL-1, and IL-6 [27]. The epidermis contains various cell types that express TLRs, including keratinocytes and Langerhans cells (LCs). In the dermis, both resident and migrating cells express TLRs. These include cells of the immune system, such as monocytes, macrophages, dendritic cells (DCs)—also known as antigen presenting cells (APCs)—and lymphocytes or mast cells [29]. The activation and maturation of APCs is induced not only by the recognition of bacterial components by PRRs such as TLRs, but also by the engagement of pro-inflammatory cytokines [30,31]. TLR4 works primarily with CD14 to recognize Gram-negative bacteria via lipopolysaccharide (LPS), although it also contributes to the recognition of Gram-positive bacteria [31,32].

In contrast, TLR2, in combination with TLR1 or TLR6, has a broader spectrum and can recognise lipoproteins as well as peptidoglycan (PGN), lipoteichoic acid (LTA), lipoarabinomannan and zymosan from Gram-positive bacteria and fungi. In addition, TLR2 and/or TLR4 can also recognize endogenous ligands or DAMPs. These include hyaluronic acid, oxidized proteins, lipids, and lipoproteins [32,33]. The immunological processes involved in the development of different forms of acne are still not clearly understood. In addition, the role of monocytes in the mechanisms of induction, inhibition and/or progression of skin lesions in untreated acne is still unknown. This study aimed to evaluate the percentage of monocyte subpopulations expressing TLR2 and TLR4, in relation to clinical data and skin function parameters, in patients with untreated acne vulgaris. Interestingly, correlation between skin hydration levels and the percentage of classical monocytes TLR2-expressing could be useful in helping to select cosmetics and skincare products for patients with acne in clinical settings. The study presents innovative and clinically relevant results. No similar scientific studies have been conducted to date.

## 2. Materials and Methods

The research used an original interview questionnaire (The questionnaire template is included in the Supplementary Materials), cytometric determinations and a device for assessing functional parameters of the skin (MPA 6 Scientific Modular Device, Courage + Khazaka). This study was conducted in accordance with the tenets of the Declaration of Helsinki. Participants were informed about the purpose and anonymity of the study and gave written informed consent to participate. Approval was granted by the Ethics Committee of the Medical University of Lublin (decision no. KE-0254/109/2021). The research was carried out as part of an internal PB grant for beginning researchers, registered under the number GW/PB/64/2021.

### 2.1. Criteria for Inclusion and Exclusion from the Study Group

The study group included 38 women with acne vulgaris. Exclusion criteria for the study were age < 18 years, gender male, symptoms of infection, diagnosed chronic disease, including autoimmune disease and cancer, and use of medications that may affect the immune system. Before the study began, each patient was informed of the purpose of the study and asked to complete an informed consent form. Women from the study group were interviewed and examined by a dermatologist, taking into account: the form of acne vulgaris, the location, type and number of skin eruptions, including non-inflammatory eruptions (closed comedones, open comedones), inflammatory eruptions (papules, pustules, cysts) and post-acne lesions (erythema, discoloration, scars), duration and course of the disease, comorbidities, including hormonal disorders, family history of acne vulgaris.

### 2.2. Assessment of Skin Function Parameters

Skin parameters were assessed using a specialised diagnostic device (Scientific multi-probe system MPA 6, Courage + Khazaka). The following probes were used to assess skin parameters: sebumeter^®^ SM 815—measurement of sebum on the skin, corneometer^®^ CM 825—measurement of skin surface hydration, mexameter^®^ MX 18—measurement of melanin and erythema, skin-pH meter PH 905—measurement of pH. Three measurements were taken on the forehead and cheek. The tests were conducted at least 8–12 h after washing the face with water and applying cosmetics, maintaining a constant temperature and relative humidity in the room as planned for each probe. Measurements were performed in accordance with the relevant guidelines to ensure good practice.

### 2.3. Isolation of Peripheral Blood Mononuclear Cells (PBMC)

Within two hours of being collected, the whole blood (5 mL) was diluted 1:1 with 0.9% PBS buffer. Then 10 mL of the diluted blood was thoroughly mixed and layered on Gradisol L (cat.no.: 9003.1, Aqua-Med, Łódź, Poland) with a specific gravity of 1.077 g/mL in a 2:1 ratio. The whole was centrifuged in a density gradient at 700× *g* for 20 min at room temperature. After 20 min of centrifugation between Gradisol L and diluted plasma, a layer of PBMC, the so-called “buffy coat”, was obtained. The mononuclear cells were then carefully collected with a Pasteur pipette and washed twice with PBS (5 min, room temperature, 700× *g*). Finally, the mononuclear cells were suspended in 1 mL of PBS and counted in a Neubauer chamber.

### 2.4. Flow Cytometry of Classical, Intermediate and Non-Classical Monocytes with Expression of TLR2 and TLR4

**Figure 1:**
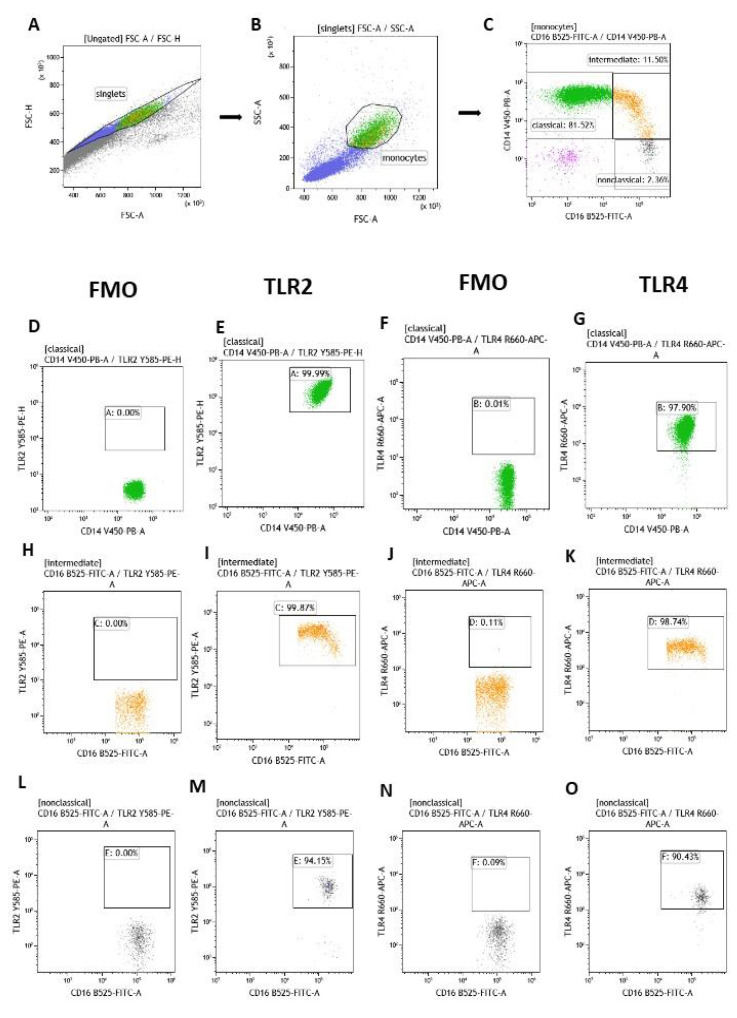
Gating strategy of monocyte subsets with TLR2 and TLR4 receptor expression in acne vulgaris patient: (**A**) Dot plot of FSC-A vs. FSC-H—target set to eliminate duplicates. (**B**) A plot of FSC vs. SSC allowed separation of monocyte populations from other PBMCs based on morphology. (**C**) Based on the expression of CD14 and CD16 surface markers, three subpopulations of monocytes were identified in the dot plot: “classical” monocytes (CD14++/CD16−) are marked in green, “intermediate” monocytes (CD14++/CD16+) are marked in orange and black for “non-classical” monocytes (CD14+/lowCD16++). A detailed assessment of the percentage of “classical”, “intermediate” and “non-classical” monocytes expressing TLR2 (**E**,**I**,**M**) and TLR4 (**G**,**K**,**O**) was performed with reference to the FMO control (**D**,**H**,**L**,**F**,**J**,**N**), which allowed correct gating of positive cells.

Isolated mononuclear cells were added to cytometric tubes (5 mL) at a concentration of 1 × 106 and incubated with appropriately selected fluorochrome-conjugated monoclonal antibodies. These were: mouse Anti-Human CD16 FITC (clone 3G8, cat.no.: 555406, BD Biosciences), Mouse Anti-Human CD14 V450 (clone: MφP9, cat.no.: 560349, BD Biosciences), mouse Anti-Human TLR2 PE (cat. no. 565349, clone: 11G7, Becton Dickinson), mouse anti-human TLR4 (CD284) APC, (cat.no. 17-9917-42, clone: HTA284 Thermo Fisher Scientific, Waltham, MA, USA) used by the manufacturer’s instructions. After 20 min incubation at room temperature in the dark, the cells were washed in PBS solution and subjected to cytometric analysis in a Cytoflex LX flow cytometer (Beckman Coulter). The control was FMO (fluorescence minus one), which allowed proper gating of the positive population and its separation from the negative population. Gating strategy is present in Figure 1. When the expression of a given molecule exceeded 95%, the mean fluorescence intensity (MFI) was assessed. MFI indirectly indicates the level of antigen expression [34].

### 2.5. Statistical Methods

The results of the research were statistically analyzed using IBM SPSS Statistics and Statistica. Statistical analyses were performed using IBM SPSS Statistics package 29. It was used to perform frequency analysis of basic descriptive statistics, Shapiro–Wilk test, Mann–Whitney U test, Spearman’s rho correlation analysis and Friedman test. The level of significance is α = 0.05.

## 3. Results

### 3.1. Characteristics of the Study Group

A total of 38 women participated in the study; the youngest respondent was 17 years old and the oldest was 33 years old, with a mean age of 21.79 ± 3.33 years. More than half of the respondents had papulopustular acne, with the presence of inflammatory eruptions (*n* = 29; 53.7%), and 31.5% (*n* = 17) had comedonal acne, with a predominance of closed or open comedones, while 4 respondents (7.4%) had keloid, where scarring occurs. 3 respondents (5.6%) had acne caused by scratching. Respondents most commonly reported acne lesions on the face (*n* = 37; 21.8%) or forehead (*n* = 31; 18.2%). Fewer women reported acne on the cheeks (*n* = 27; 15.9%) or chin (*n* = 24; 14.1%). Most women reported papules, pustules, cysts, i.e., the inflammatory nature of the eruptions (27 respondents, 71.1%), while the non-inflammatory nature of the eruptions was reported by 17 women (32.1%). However, at the time of the study, 19.6% (*n* = 11) of the respondents had post-acne lesions. The vast majority of respondents had moderate acne vulgaris (*n* = 22; 57.9%). 13 women (34.2%) had mild symptoms, while 7.9% of respondents (*n* = 3) had severe disease.

For the sebumeter measurements, a greater percentage of respondents obtained results within the norm (52.6% for the forehead and 60.5% for the cheek). In the case of the forehead corneometer result, the vast majority (78.9%) obtained a result indicating adequately moisturised skin, while on the cheek, most people (42.1%) had a result indicating dry skin. The phototype measured with a mexameter was type II in the majority of respondents (63.2% for the forehead and 52.6% for the cheek). None of the subjects obtained results indicating phototype IV, V or VI. In both forehead and cheek measurements, the results indicated the presence of slight erythema in most people (57.9% for forehead and 65.8% for cheek). The pH was high in almost all subjects (94.7%) for the forehead and all subjects (100.0%) for the cheek. None of the subjects had a pH reading within the normal range (Table 1).

### 3.2. Comparison of Skin Functional Parameters and Percentages of Classical, Intermediate and Non-Classical Monocytes with TLR2 and TLR4 Expression

The results of the analysis of phototype, measured on the forehead and cheeks, and erythema, measured on the forehead and cheeks with a mexameter, were statistically significantly lower in people who had post-acne lesions compared with people who did not have such changes.

The level of oiliness measured with a sebumeter was significantly higher in people with acne lesions. Skin hydration on the forehead and cheeks was also significantly higher in people with acne lesions than in people without erythema, discolouration or scarring. The pH levels remained relatively similar. It is important to use properly selected home care products and carry out cosmetological procedures in the long term, both during the development of acne lesions, during treatment, and after the dermatosis has disappeared. In the group of patients with post-acne lesions, a statistically significantly higher percentage of non-classical monocytes expressing TLR4 was observed compared to patients without post-acne lesions (i.e., erythema, scars) (*p* = 0.009). In addition, the level of TLR4 expression on classical and intermediate monocytes was statistically significantly higher in the group of patients with post-acne lesions compared to the level of expression of this receptor on the analyzed subpopulations of monocytes in the group of patients without post-acne lesions. It suggests an important role for these monocyte subpopulations in maintaining inflammation in the long-term course of acne. Such data may suggest that monocytes expressing TLR4 may be most susceptible to activation in the long-term inflammatory response induced by *C. acnes* (Table 2).

The results of the analysis showed that people with papulopustular acne with inflammatory eruptions had higher skin functional parameters in terms of oiliness (forehead, cheek), hydration (forehead, cheek), erythema (forehead, cheek), and pH (forehead) compared to patients who did not suffer from the inflammatory form of papulopustular acne. Higher scores for skin oiliness, especially erythema, and pH may indicate a disordered hydrolipidic barrier. A disturbance in the microbiological balance of the skin favors the development of inflammation and prolongs the process of skin regeneration. Appropriate dermatological and cosmetological treatment is required. A cosmetologist can help you choose products and treatments that reduce skin hypersensitivity and strengthen the skin’s protective barrier (Table 3).

### 3.3. Correlations Between the Percentage of Individual Monocyte Subpopulations with TLR2 and TLR4 Expression and Functional Skin Parameters

The results showed a weak negative correlation between TLR2 expression on classical monocytes and corneometer measurement on the forehead (rho = −0.35, *p* = 0.029) and cheek (rho = −0.34, *p* = 0.036). However, a positive, statistically significant correlation was observed between the results of the cheek corneometer measurement and the percentage of classical monocytes expressing TLR2 (rho = 0.38, *p* = 0.020), whereas in this group, a negative, statistically significant correlation was observed with the percentage of classical monocytes expressing TLR4 (rho = −0.41, *p* = 0.011). Furthermore, a statistically significant negative correlation was found between the percentage of classical monocytes expressing TLR4 and the phototype measurement using a mexameter on the cheek (rho = −0.37, *p* = 0.024) (Table 4).

The results obtained showed a weak negative correlation between the level of TLR2 expression on intermediate monocytes and the results of the corneometer measurement on the forehead (*rho* = −0.33, *p* = 0.042) in patients with acne vulgaris. In addition, a statistically significant negative correlation was observed between the percentage of intermediate monocytes with TLR4 expression and the results of corneometer measurements on the cheek (*rho* = −0.39, *p* = 0.014) and on the forehead (*rho* = −0.38, *p* = 0.020) (Table 5).

In addition, a negative correlation was found between TLR2 expression on non-classical monocytes and the results of the forehead corneometer measurement (rho = −0.45, *p* = 0.005). In addition, a statistically significant negative correlation was found between the percentage of non-classical monocytes expressing TLR4 and the phototype measurement using a mexameter on the forehead (rho = −0.37, *p* = 0.024), cheek (rho = −0.35, *p* = 0.032) and erythema—forehead (rho = −0.41, *p* = 0.010).

Interestingly, correlations between the parameters measured by a corneometer and a mexameter were observed for the monocyte subpopulations studied. The results may indicate the need to use moisturizing, soothing, calming, and vasodilatory products in the course of acne vulgaris, which is often associated with persistent inflammation. Proper cosmetological care can influence the duration and effectiveness of inflammatory and non-inflammatory lesions on the skin and alleviate existing skin problems. Holistic care of a patient with acne vulgaris is also necessary, including the care of specialists in many fields, such as a dietician and an endocrinologist. It is important to correctly identify the causes of skin problems and to implement a multidisciplinary treatment. This is all the more important as the cosmetic market increasingly encourages the use of several or even a dozen anti-acne products and cosmetological treatments, promising unrealistic results (Table 6).

The results of the analysis were found to be insignificant for all variables tested, indicating that there were no differences between those with mild acne severity and those with moderate to severe acne severity (Table 7).

## 4. Discussion

The results of our research showed the need for further analysis to assess the relationship between the immune system and acne vulgaris. The influence of peripheral blood monocyte subpopulations in the pathogenesis of various forms of acne remains unclear. However, we have demonstrated differences in the percentage of classical, intermediate, and non-classical monocyte subpopulations expressing TLR2 and TLR4 in patients with untreated acne vulgaris with selected skin parameters. The percentage of classical monocytes expressing TLR2 measured “ex vivo” showed a significant positive correlation with the assessment of changes in skin hydration on the cheek measured with a corneometer. In turn, the percentage of all monocyte subpopulations expressing TLR4 correlated negatively with parameters assessed by a mexameter, especially on the cheek, i.e., erythema and melanin level. Therefore, clinicians should educate acne patients not only about diet and exposure to skin-irritating allergens but also about skin care methods, the type of cosmetics used, and everyday products, as these aspects turned out to be significantly correlated with the percentages of classical intermediate and non-classical monocytes with TLR2 and TLR4 expression. The study presents innovative and clinically relevant results. Due to the difficulties in understanding the mechanisms involved in the development of acne, Tang et al. [35] developed an in vitro acne disease model that mimics the inflammatory environment of acne lesions in the skin. The scientists generated artificial sebocyte glands and 3D macrophages and showed that sebocyte glands respond to *C. acnes* by release antimicrobial peptides and proinflammatory cytokines, including IL-1, TNF-α, IL-8, and IL-12, with the involvement of TLR2 and TLR4 receptor-dependent signaling [35]. Recently, there have been increasing reports on the impact of unbalanced activation of TLRs in triggering or exacerbating inflammatory skin diseases, including atopic dermatitis and even skin cancer and autoimmune diseases (i.e., type 1 diabetes, rheumatoid arthritis, psoriasis) [36,37,38].

Macrophages, which originate from monocytes, have an ambiguous role in the pathogenesis of acne, on the one hand being guardians, on the other hand, being provocateurs of inflammation [26]. TLR2 has been shown to be highly expressed on perifollicular and peribulbar macrophages in acne lesions, and then in activation of the signaling cascade NLRP3 inflammasome, NF-kB and MAPK signalling pathways [39,40].

Ozlu et al. showed that TLR2 and TLR4, determined by immunohistochemistry from skin biopsy material of patients with acne vulgaris, showed increased expression of TLR2 and TLR4 in the epidermis, especially in keratinocytes of patients with inflammatory, pustular clinical manifestations of acne [41]. However, the authors did not assess the functional state of the skin or its “level of care” before the biopsy. In our research, we wanted to assess the level of expression of TLR2 and TLR4 on monocyte subpopulations in acne patients in the context of assessing the state of skin hydration, oiliness, pH, and erythema intensity using specialized probes. This approach may, in the future, make it easier for dermatologists and cosmetologists to quickly assess a patient’s chances of alleviating the clinical symptoms of acne with acne skin care products by monitoring not only the appearance of the skin but also the percentage of TLR2 and TLR4 monocyte subpopulations in the patient’s blood. In 2022, Lee H. et al. observed the impact of various treatments, including microcurrent stimulation, on the degree of inhibition or stimulation of TLR2-dependent pathways [42]. Moreover, Dispenza et al. showed that isotretinoin treatment reduced the expression of TLR2 on monocytes, but did not affect TLR4 expression. This suggests that reduced TLR2 expression may be an important mechanism in the treatment of acne with isotretinoin [43]. Our research is pioneering and innovative, as it concerns the advanced cosmetological assessment of skin parameters (hydration, oiliness, pH, erythema, and phototype) in acne patients in the context of differences in the expression level of TLR2 and TLR4 on monocyte subpopulations measured by flow cytometry.

The present study has several limitations, such as a small group, only female patients included, and a lack of a control group. Due to the small size of our study group, we focused on creating a homogeneous group in terms of gender to minimise the risk of additional factors affecting the variability of immune parameters. Acne vulgaris in men is often more severe, more resistant to treatment, and more likely to cause scarring. Our research was cross-sectional and preliminary in nature and concerned only one group, in order to verify whether our hypothesis made sense and whether it was worth designing a larger study that would also include a control group.

## 5. Conclusions

Monocyte subpopulations expressing TLR2 and TLR4 circulating in the peripheral blood of patients with acne vulgaris appear to be indifferent to altered skin parameters: level of oiliness, hydration, and skin pigmentation (phototype, erythema). Furthermore, a significantly higher percentage of non-classical TLR4+ monocytes was observed in the blood of acne patients with visible post-acne lesions compared to patients without post-acne cutaneous complications. In addition, the positive correlation between the level of skin hydration and the level of TLR2 expression on classical monocytes suggests their crucial role in skin homeostasis and defense against *C. acnes*. Furthermore, proper acne care is not only important for aesthetic reasons but also for immunological changes.

## Figures and Tables

**Table 1:** Characteristics of the study group in terms of sebum secretion, hydration, skin phototype, erythema and pH assessed on the forehead and cheek.

Skin Parameter	Location	Range	*n*	%
Sebumeter— Secretion of sebum	forehead	<100—dry skin, less sebum	17	44.7%
		100–220—normal	20	52.6%
		>220—oily skin	1	2.6%
		Total	38	100.0%
	cheek	<70—dry skin, less sebum	14	36.8%
		70–180—normal	23	60.5%
		>180—oily skin	1	2.6%
		Total	38	100.0%
Corneometer— Hydration	forehead	<30—very dry skin	1	2.6%
		30–45—dry skin	7	18.4%
		>45—skin sufficiently moisturized	30	78.9%
		Total	38	100.0%
	cheek	<30—very dry skin	9	23.7%
		30–45—dry skin	16	42.1%
		>45—skin sufficiently moisturized	13	34.2%
		Total	38	100.0%
Mexameter—phototype	forehead	0–100—Photo type I (Nordic/Celtic skin)	10	26.3%
		100–150—Photo type II (Light Caucasians)	24	63.2%
		150–250—Photo type III (European mixed type/very fair Asian skin)	4	10.5%
		250–350 Photo type IV (Mediterranean/fari Asian skin)	0	0.0%
		350–450 Photo type V (Dark skin)	0	0.0%
		>450 Photo type VI (Black skin)	0	0.0%
		Total	38	100.0%
Mexameter—phototype	cheek	0–100—Photo type I (Nordic/Celtic skin)	15	39.5%
		100–150—Photo type II (Light Caucasians)	20	52.6%
		150–250—Photo type III (European mixed type/very fair Asian skin)	3	7.9%
		250–350 Photo type IV (Mediterranean/fari Asian skin)	0	0.0%
		350–450 Photo type V (Dark skin)	0	0.0%
		>450 Photo type VI (Black skin)	0	0.0%
		Total	38	100.0%
Mexameter—erythema	forehead	0–170—no erythema	3	7.9%
		170–330—minimal erythema	22	57.9%
		330–450—diffuse erythema	12	31.6%
		450–570—high erythema	1	2.6%
		>570—extreme erythema	0	0.0%
		Total	38	100.0%
Mexameter—erythema	cheek	0–170—no erythema	1	2.6%
		170–330—minimal erythema	25	65.8%
		330–450—diffuse erythema	8	21.1%
		450–570—high erythema	4	10.5%
		>570—extreme erythema	0	0.0%
		Total	38	100.0%
Skin-pH-Meter—pH	forehead	+ acidic range-	2	5.3%
		normal	0	0.0%
		− high skin pH value+	36	94.7%
		Total	38	100.0%
Skin-pH-Meter—pH	cheek	+ acidic range −	0	0.0%
		normal	0	0.0%
		− high skin pH value +	38	100.0%
		Total	38	100.0%

Annotation. *n*—number of observations. The range has been included in accordance with the instructions of the eprus device distributor/manufacturer, COURAGE + KHAZAKA Electronic GmbH.

**Table 2:** Analysis of parameters measured using a sebumeter, corneometer, mexameter and pH, and the percentage of three monocyte subpopulations (classical, intermediate, non-classical) expressing TLR2 and TLR4 in patients with acne vulgaris with or without post-acne lesions.

	Occurrence of Post-Acne Changes (Erythema, Discoloration, Scars)						Z	*p*
	Yes (*n* = 11)			No (*n* = 27)				
	M	Me	SD	M	Me	SD		
Sebumeter—forehead	129.64	143.00	54.75	106.00	95.00	49.39	−1.22	0.22
Sebumeter—cheek	102.82	93.00	42.31	82.22	72.00	41.78	−1.37	0.17
Corneometer—forehead	53.94	53.67	9.53	51.39	52.30	11.47	−0.82	0.41
Corneometer—cheek	41.65	39.23	11.67	37.57	35.77	13.29	−0.76	0.45
Mexameter- phototype—forehead	90.64	85.33	25.62	126.84	124.00	29.82	−3.25	0.00
Mexameter- phototype—cheek	85.17	79.00	23.32	108.86	106.33	31.16	−2.40	0.02
Mexameter- erythema—forehead	234.59	221.67	56.16	312.67	319.00	78.65	−2.86	0.00
Mexameter- erythema—cheek	259.26	226.23	68.31	327.77	316.33	93.76	−2.17	0.03
pH—forehead	6.96	6.56	2.57	5.85	5.87	0.97	−1.38	0.17
pH—cheek	6.05	5.20	1.41	6.11	5.98	1.11	−0.50	0.62
Classical—CD14++CD16-	86.05	86.39	6.14	86.53	86.80	5.50	−0.18	0.86
Classical with TLR2 (%)	96.86	97.62	3.58	97.35	98.93	4.47	−0.69	0.49
Classical TLR2 (MFI)	176,639.02	159,580.48	58728.15	189,566.68	194,171.20	64,745.11	−0.47	0.64
Classical with TLR4 (%)	97.24	98.31	3.01	94.59	96.73	7.06	−1.01	0.31
Classical TLR4 (MFI)	8545.39	6217.96	8979.67	45,942.43	3747.89	215,344.42	−2.72	0.01
Intermediate—CD14++CD16+	6.72	6.44	2.54	6.22	5.86	2.76	−0.69	0.49
Intermediate with TLR2 (%)	96.07	97.24	3.61	96.24	98.89	10.93	−1.32	0.19
Intermediate TLR2 (MFI)	407,542.37	258,617.46	371,861.53	574,693.01	265,604.26	1,121,976.14	−0.34	0.74
Intermediate with TLR4 (%)	91.69	93.00	2.96	86.70	88.02	11.69	−0.85	0.39
Intermediate TLR4 (MFI)	7758.55	7987.94	2174.22	6138.33	5177.02	2753.74	−2.43	0.02
Non-classical—CD14+/lowCD16^++^	2.64	2.55	1.02	2.51	2.02	1.25	−0.95	0.34
Non-classical with TLR2 (%)	89.56	91.42	8.96	92.18	93.12	5.81	−0.69	0.49
Non-classical TLR2 (MFI)	204,353.42	160,257.39	134,941.57	721,003.20	171,728.03	2,646,051.93	−0.34	0.74
Non-classical with TLR4 (%)	94.51	94.33	3.20	87.16	89.77	9.92	−2.62	0.01
Non-classical TLR4 (MFI)	10,675.59	5606.41	15,948.57	6652.76	4784.17	5087.21	−1.69	0.09

Annotation. M—mean; Me—median; SD—standard deviation; *p*—statistical significance. *p* < 0.05, MFI—mean fluorescence intensity.

**Table 3:** Analysis of parameters measured using a sebumeter, corneometer, mexameter and pH and the percentage of three monocyte subpopulations (classical, intermediate, non-classical) expressing TLR2 and TLR4 in patients with and without papulopustular acne with inflammatory eruptions.

	The Occurrence of Papulopustular Acne, with the Presence of Inflammatory Eruptions						Z	*p*
	Yes (*n* = 29)			No (*n* = 9)				
	M	Me	SD	M	Me	SD		
Sebumeter—forehead	114.76	117.00	50.38	106.67	79.00	57.28	−0.70	0.48
Sebumeter—cheek	91.60	88.00	43.66	77.15	66.33	38.43	−0.76	0.45
Corneometer—forehead	52.94	53.47	11.52	49.51	50.90	8.53	−1.18	0.24
Corneometer—cheek	38.84	39.23	14.33	38.49	37.27	6.48	−0.02	0.99
Mexameter- phototype—forehead	114.01	118.67	30.35	123.93	113.67	41.01	−0.05	0.96
Mexameter- phototype—cheek	104.92	105.23	26.88	92.59	85.00	41.54	−0.74	0.46
Mexameter- erythema—forehead	298.97	279.33	72.51	261.37	247.67	101.91	−1.25	0.21
Mexameter- erythema—cheek	308.51	297.50	86.25	306.07	263.00	113.79	−0.22	0.82
pH—forehead	6.27	5.99	1.83	5.84	5.61	0.82	−0.60	0.55
pH—cheek	6.08	5.98	1.27	6.11	5.69	0.91	−0.22	0.82
Classical-CD14++CD16-	86.20	86.60	5.92	87.00	88.38	4.72	−0.09	0.93
Classical with TLR2 (%)	96.66	97.90	4.65	98.98	99.42	1.03	−1.60	0.11
Classical TLR2 (MFI)	188,518.70	191,080.59	67,725.44	177,143.02	194,171.20	43,972.91	−0.26	0.80
Classical with TLR4 (%)	94.93	97.40	6.95	96.74	98.04	2.81	−0.15	0.88
Classical TLR4 (MFI)	44,798.76	4469.56	207,532.37	3920.10	3683.52	1220.89	−1.60	0.11
Intermediate—CD14++CD16+	6.47	6.03	2.74	6.02	5.86	2.59	−0.39	0.69
Intermediate with TLR2 (%)	95.49	98.57	10.62	98.46	98.89	1.55	−0.62	0.54
Intermediate TLR2 (MFI)	600,954.50	265,604.26	109,4922.39	285,777.42	253,970.75	100,338.78	−0.98	0.33
Intermediate with TLR4 (%)	87.03	89.71	11.04	91.75	93.51	5.82	−1.12	0.26
Intermediate TLR4 (MFI)	6834.91	5830.45	2756.43	5874.07	5177.02	2394.47	−1.08	0.28
Non-classical—CD14+/lowCD16++	2.50	2.02	1.27	2.71	2.66	0.83	−0.77	0.44
Non-classical with TLR2 (%)	90.17	91.42	7.12	95.44	95.29	3.98	−2.35	0.02
Non-classical TLR2 (MFI)	696,514.14	162,954.85	255,2202.16	168,451.55	162,931.91	35,061.29	−0.26	0.80
Non-classical with TLR4 (%)	89.04	92.48	10.11	90.09	90.95	5.15	−0.46	0.64
Non-classical TLR4 (MFI)	8433.55	5240.34	10,660.78	5831.45	4431.87	3777.49	−1.29	0.20

Note. *n*—number of observations; M—mean; Me—median; SD—standard deviation; Z—value of the test statistic; *p*—statistical significance.

**Table 4:** Results of the correlation analysis between the measurements made with the sebumeter, corneometer, mexameter, pH and the percentage of classical monocyte subpopulations CD14++CD16-TLR2+, CD14++CD16-TLR4+, and the expression level of the tested receptors (MFI) on classical monocytes in the study group.

		Classical—CD14++CD16-	Classical with TLR2 (%)	Classical TLR2 (MFI)	Classical with TLR4 (%)	Classical TLR4 (MFI)
Sebumeter—forehead	*rho*	0.14	−0.02	−0.07	0.22	0.10
	*p*	0.41	0.90	0.66	0.18	0.57
Sebumeter—forehead	*rho*	0.15	−0.08	−0.08	−0.14	−0.19
	*p*	0.36	0.62	0.63	0.40	0.26
Corneometer—forehead	*rho*	0.04	0.06	−0.35	−0.13	−0.03
	*p*	0.80	0.72	0.03	0.43	0.86
Corneometer—cheek	*rho*	−0.05	0.38	−0.34	−0.41	0.02
	*p*	0.76	0.02	0.04	0.01	0.91
Mexameter- phototype—forehead	*rho*	0.00	0.15	0.24	−0.26	−0.27
	*p*	1.00	0.37	0.15	0.12	0.11
Mexameter- phototype—cheek	*rho*	−0.14	0.18	0.09	−0.37	−0.31
	*p*	0.41	0.29	0.58	0.02	0.06
Mexameter- erythema—forehead	*rho*	0.10	−0.09	0.30	−0.17	−0.27
	*p*	0.55	0.58	0.07	0.30	0.10
Mexameter- erythema—cheek	*rho*	0.29	−0.07	0.00	0.14	−0.13
	*p*	0.08	0.68	0.98	0.40	0.44
pH—forehead	*rho*	−0.04	0.20	−0.20	−0.26	0.25
	*p*	0.82	0.22	0.22	0.12	0.13
pH—cheek	*rho*	−0.03	0.25	0.11	−0.21	0.10
	*p*	0.84	0.13	0.51	0.21	0.54

**Table 5:** Results of the correlation analysis between measurements performed using a sebumeter, corneometer, mexameter, pH and the percentage of intermediate monocyte subpopulations—CD14++CD16+TLR2+, CD14++CD16+TLR4+—taking into account the expression level of TLR2 and TLR4 (MFI) on intermediate monocytes.

		Intermediate CD14++CD16+	Intermediate z TLR2 (%)	Intermediate TLR2 (MFI)	Intermediate with TLR4 (%)	Intermediate TLR4 (MFI)
Sebumeter—forehead	*rho*	0.05	−0.08	−0.07	0.06	0.12
	*p*	0.76	0.64	0.66	0.71	0.47
Sebumeter—forehead	*rho*	−0.10	0.01	−0.04	−0.07	−0.12
	*p*	0.54	0.97	0.79	0.69	0.47
Corneometer—forehead	*rho*	0.25	0.01	−0.33	−0.26	−0.07
	*p*	0.12	0.94	0.04	0.11	0.68
Corneometer—cheek	*rho*	0.20	0.15	−0.21	−0.24	−0.16
	*p*	0.22	0.38	0.20	0.14	0.35
Mexameter- phototype—forehead	*rho*	0.04	0.20	0.14	−0.29	−0.23
	*p*	0.80	0.23	0.41	0.08	0.17
Mexameter- phototype—cheek	*rho*	0.06	0.20	0.07	−0.39	−0.18
	*p*	0.72	0.23	0.67	0.01	0.29
Mexameter- erythema—forehead	*rho*	−0.15	0.06	0.21	−0.38	−0.24
	*p*	0.38	0.73	0.21	0.02	0.15
Mexameter- erythema—cheek	*rho*	−0.22	0.01	−0.02	−0.01	−0.17
	*p*	0.19	0.98	0.92	0.98	0.30
pH—forehead	*rho*	0.16	0.09	−0.14	0.20	0.06
	*p*	0.33	0.60	0.41	0.22	0.71
pH—cheek	*rho*	0.13	0.16	0.04	0.16	0.13
	*p*	0.43	0.34	0.83	0.34	0.44

**Table 6:** Results of the correlation analysis between measurements performed using a sebumeter, corneometer, mexameter, pH and the percentage of non-classical monocyte subpopulations—CD14+/lowCD16++TLR2+, CD14+/lowCD16++TLR4+—taking into account the expression level of TLR2 and TLR4 (MFI) on non-classical monocytes.

		Non-Classical CD14+/lowCD16++	Non-Classical with TLR2 (%)	Non-Classical TLR2 (MFI)	Non-Classical with TLR4 (%)	Non-Classical TLR4 (MFI)
Sebumeter—forehead	*rho*	−0.05	0.08	−0.18	0.09	−0.10
	*p*	0.77	0.62	0.29	0.59	0.54
Sebumeter—forehead	*rho*	0.01	0.00	−0.05	−0.20	−0.25
	*p*	0.94	1.00	0.78	0.23	0.13
Corneometer—forehead	*rho*	0.00	0.03	−0.45	−0.03	−0.20
	*p*	0.98	0.88	0.01	0.85	0.24
Corneometer—cheek	*rho*	−0.20	−0.06	0.06	−0.13	0.01
	*p*	0.22	0.71	0.71	0.43	0.97
Mexameter- phototype—forehead	*rho*	−0.20	−0.23	0.20	−0.37	−0.07
	*p*	0.22	0.17	0.23	0.02	0.67
Mexameter- phototype—cheek	*rho*	−0.13	−0.15	0.15	−0.35	−0.11
	*p*	0.45	0.38	0.38	0.03	0.51
Mexameter- erythema—forehead	*rho*	−0.27	−0.22	0.05	−0.41	−0.13
	*p*	0.10	0.20	0.75	0.01	0.44
Mexameter- erythema—cheek	*rho*	−0.19	−0.08	−0.14	−0.20	−0.18
	*p*	0.26	0.62	0.41	0.24	0.28
pH—forehead	*rho*	0.26	0.10	0.10	0.32	0.14
	*p*	0.12	0.57	0.53	0.05	0.40
pH—cheek	*rho*	−0.02	−0.05	0.13	0.20	0.18
	*p*	0.89	0.77	0.43	0.23	0.27

**Table 7:** Analysis of parameters measured using a sebumeter, corneometer, mexameter and pH and the percentage of three monocyte subpopulations (classical, intermediate, non-classical) expressing TLR2 and TLR4 in patients with varying degrees of severity and course of acne.

	Severity of Acne						Z	*p*
	Mild (*n* = 13)			Medium Severity/Moderate or Severe (*n* = 25)				
	M	Me	SD	M	Me	SD		
Sebumeter—forehead	103.08	79.00	45.61	117.92	117.00	54.36	−0.95	0.34
Sebumeter—cheek	70.72	59.00	39.06	97.26	93.00	41.97	−1.89	0.06
Corneometer—forehead	50.14	53.63	14.21	53.16	52.30	8.85	−0.11	0.91
Corneometer—cheek	36.76	35.63	14.33	39.79	39.23	12.15	−0.54	0.59
Mexameter- phototype—forehead	122.95	120.33	22.72	112.93	112.00	37.01	−0.97	0.33
Mexameter- phototype—cheek	96.74	104.67	31.20	104.73	105.23	30.85	−0.40	0.69
Mexameter- erythema—forehead	308.90	321.67	94.55	280.27	271.00	72.37	−1.12	0.26
Mexameter- erythema—cheek	324.56	282.67	101.93	299.29	297.50	87.03	−0.75	0.45
pH—forehead	5.90	5.88	0.72	6.31	5.99	1.96	−0.40	0.69
pH—cheek	5.85	5.69	0.76	6.22	6.04	1.35	−0.89	0.37
Classical—CD14++CD16-	86.78	87.92	5.75	86.19	86.60	5.65	−0.14	0.89
Classical with TLR2 (%)	97.89	98.13	2.42	96.85	98.56	4.88	−0.08	0.94
Classical TLR2 (MFI)	193,675.25	198,579.74	82,354.79	181,742.05	185,268.68	50,900.51	−0.23	0.82
Classical with TLR4 (%)	95.89	96.73	3.89	95.08	97.82	7.23	−0.08	0.94
Classical TLR4 (MFI)	5333.95	4234.98	3473.94	50,604.14	4469.56	223,585.20	−0.42	0.68
Intermediate—CD14++CD16+	5.87	5.86	2.71	6.62	6.03	2.68	−0.85	0.40
Intermediate with TLR2 (%)	94.45	99.08	15.66	97.09	98.45	3.13	−0.91	0.36
Intermediate TLR2 (MFI)	758,851.56	265,604.26	1,589,525.36	405,384.28	261,178.94	350,185.74	−0.42	0.68
Intermediate with TLR4 (%)	89.12	93.51	9.93	87.64	90.02	10.48	−0.75	0.45
Intermediate TLR4 (MFI)	5942.71	5083.32	2372.85	6952.95	5910.13	2803.69	−1.62	0.11
Non-classical— CD14+/lowCD16++	2.34	2.45	0.74	2.66	2.05	1.35	−0.68	0.50
Non-classical with TLR2 (%)	92.59	93.43	6.42	90.81	92.24	7.11	−0.91	0.36
Non-classical TLR2 (MFI)	256,187.73	154,627.26	364,869.10	735,381.34	171,728.03	2,744,298.89	−0.63	0.53
Non-classical with TLR4 (%)	89.83	90.95	5.95	89.01	91.38	10.51	−0.48	0.63
Non-classical TLR4 (MFI)	5913.00	4784.17	3259.41	8807.48	5240.34	11,445.13	−0.88	0.38

## Data Availability

The data generated and analysed during this study are available from the corresponding authors upon reasonable request.

## Supplementary Materials

The following supporting information can be downloaded at https://www.mdpi.com/article/10.3390/jcm14186449/s1. Final version of Interview Questionnaire.
