# Supplementary material for: Evaluation of the Percentage of Monocyte Subpopulations with TLR2 and TLR4 Expression About Selected Skin Functional Parameters in Patients with Acne Vulgaris—Cross-Sectional Study
Source: J Clin Med. 2025 Sep 12;14(18):6449. doi: 10.3390/jcm14186449 (PMC12470757; doi:10.3390/jcm14186449)
Supplement: Supplementary file 1 [file jcm-14-06449-s001.zip › jcm-3831801-supplementary.pdf]

Supplementary Materials

INTERVIEW QUESTIONNAIRE

1. Age:

……………………

2. Gender:

A. female

B. male

3. Place of residence:

A. rural area

B. urban area

4. Education:

A. higher education

B. secondary education

C. vocational education

D. primary education

5. Social and living conditions:

A. very good

B. good

C. average

D. poor

E. very poor

6. How would you rate your health?

A. very good

B. good

C. average

D. poor

E. very poor

INTERVIEW AND CLINICAL EXAMINATION/ASSESSMENT
DERMATOLOGICAL − COSMETOLOGICAL
(to be completed by a specialist)

1. Form of common acne:

A. comedonal, with a predominance of closed and/or open comedones

B. papulopustular, with the presence of inflammatory lesions

C. pustular

D. cicatricial, with scarring

E. fulminant

F. acne excoriata

2. Duration of acne:

………………….. months/years

3. Location of acne lesions:

A. Face

B. Forehead

C. Nose

D. Cheeks

E. Chin

F. Neck

G. Neckline

H. Back

I. Other ………………………

4. Nature of skin lesions:

A. Non-inflammatory (closed comedones, open comedones),

B. Inflammatory (papules, pustules, cysts)

C. Acne scars (erythema, hyperpigmentation, scars),

5. Severity and course of acne vulgaris:

A. Mild

B. Moderate

C. Severe

6. Family history of acne vulgaris:

A. Positive

B. Negative

C. No data available

7. Dermatological medications applied externally to the skin:

A. Yes (which ones?)

B. No

8. Oral dermatological medications:

A. Yes (which ones?)…………………………………………………………………

B. No

9. Comorbidities, including hormonal disorders:

A. Yes (which ones?)………………………………………………………………

B. No

10. Other chronic skin conditions?

A. Yes (which ones?)…………………………………………………………………

B. No
